# Advancing AI-based clinical decision support for complex interventions requires an operationalized framework

**DOI:** 10.1097/PR9.0000000000001397

**Published:** 2026-01-16

**Authors:** Björn Äng, Tony Bohman, Anna Grimby-Ekman, Johan Ärnlöv, Ilias Thomas, Roger Nyberg, Elena Tseli, Linda Vixner, Marika Hagelberg, Riccardo LoMartire

**Affiliations:** a School of Health and Welfare, Dalarna University, Falun, Sweden; b Department of Research, Education and Innovation, Center for Clinical Research Dalarna, Uppsala University, Region Dalarna, Falun, Sweden; c Division of Physiotherapy, Department of Neurobiology, Care Sciences and Society, Karolinska Institutet, Huddinge, Sweden; d Department of Physical Education and Sport Science, Biomechanical and Ergonomic Laboratory, University of Thessaly, Trikala, Greece; e Department of Women's and Children's Health, Physiotherapy and Behavioral Medicine, Uppsala University, Uppsala, Sweden; f GothPain, School of Public Health and Community Medicine, Institute of Medicine, University of Gothenburg, Gothenburg, Sweden; g Division of Family Medicine and Primary Care, Department of Neurobiology, Care Sciences and Society, Karolinska Institutet, Huddinge, Sweden; h School of Information and Engineering, Dalarna University, Borlange, Sweden

## Letter to the Editor:

Chronic pain is one of the leading causes of global disability and lost productive life years,^3^ placing a substantial burden on individuals and healthcare systems.^2^ This burden is further intensified by longer life expectancy—a positive trend that, while welcome, also contributes to population ageing and the rising prevalence of chronic disease.^3^ Interdisciplinary treatment (IDT), a complex intervention defined by multiple interacting components, integrates physical activity, education, cognitive–behavioral therapy, and pharmacological strategies.^2^ It remains the most comprehensive approach for managing chronic pain.^2^ However, its long-term effectiveness remains uncertain. Even highly experienced IDT teams face challenges in patient selection and individualized care, and clinical practices continue to vary substantially across settings.

AI-based clinical decision support systems (AI-CDSS), with their ability to process large and multifaceted data sets through modern AI-driven approaches,^1^ hold strong promise for addressing this complexity and variability in IDT.^4^ By integrating historical data, trial results, and real-world evidence, AI-CDSS can generate insights that support shared goal setting between patients and interdisciplinary teams, while also providing individualized probabilities of treatment success. In this way, they can strengthen personalized IDT and advance precision in pain management by moving beyond one-size-fits-all approaches and aligning care more closely with both scientific evidence and patient-defined priorities.^1^ However, current development remains constrained by limited real-world data sets, insufficient external validation,^1^ and persistent concerns over transparency and algorithmic bias.^1,4^

The updated Medical Research Council (MRC) guidance outlines useful principles for phased development, feasibility testing, and evaluation of complex interventions.^5^ It does not, however, fully capture the adaptive, iterative, and data-driven processes required as AI-based systems evolve in health care. Reflecting this gap, recent work in pain medicine has proposed methodological frameworks for structured AI development,^1^ although these still overlook the unique challenges of IDT as a complex intervention for chronic pain. As AI-based CDSS advance rapidly in health care, the urgency of an extended framework becomes evident. Key challenges—bias, explainability, and workflow integration—define the conditions for safe and effective AI-CDSS. Without systematic validation across diverse populations, AI risks amplifying inequities rooted in historical data^1^; without explainability, models operate as opaque “black boxes,” eroding trust among clinicians and patients; and without adequate training and workflow adaptation, AI may displace rather than support clinical reasoning. These challenges must be embedded as mandatory checkpoints in a structured developmental pathway, not treated as optional add-ons.

We argue that an operationalized framework, extending the established MRC guidance, is essential to ensure that AI-CDSS for *complex interventions* are developed with scientific rigor, equity, and clinical trustworthiness. Such a framework would provide a phased roadmap—from early validation and stakeholder engagement to scalability and sustainability—so that rapid technological advances remain aligned with the realities and practice. Proactively adopted, it could transform AI-CDSS into a true enabler of personalized and precision-oriented care in chronic pain and other complex conditions, rather than a disruptive force that risks delaying clinical translation for another decade.

## Disclosures

J.Ä. has received lecturing fees from AstraZeneca and Boehringer Ingelheim and has served on advisory boards for AstraZeneca, Astellas, and Boehringer Ingelheim. These activities are unrelated to the present article. All other authors declare no conflicts of interest.
